# Insights into bacterial CO_2_ metabolism revealed by the characterization of four carbonic anhydrases in *Ralstonia eutropha* H16

**DOI:** 10.1186/2191-0855-4-2

**Published:** 2014-01-10

**Authors:** Claudia S Gai, Jingnan Lu, Christopher J Brigham, Amanda C Bernardi, Anthony J Sinskey

**Affiliations:** 1 Department of Biology, Massachusetts Institute of Technology, Bldg. 68-370, Cambridge, MA 02139, USA; 2 Department of Chemistry, Massachusetts Institute of Technology, 77 Massachusetts Avenue, Cambridge, MA 02139, USA; 3 Department of Bioengineering, University of Massachusetts Dartmouth, 285 Old Westport Road, North Dartmouth, MA 02747, USA; 4 Division of Health Sciences and Technology, Massachusetts Institute of Technology, 77 Massachusetts Avenue, Bldg. 68-370, Cambridge, MA 02139, USA; 5 Engineering Systems Division, Massachusetts Institute of Technology, 77 Massachusetts Avenue, Bldg. 68-370, Cambridge, MA 02139, USA

**Keywords:** CO_2_ transport, *Cupriavidus necator*, Carbon dioxide, Bicarbonate, Dissolved inorganic carbon, Periplasm, Zinc metalloenzyme

## Abstract

Carbonic anhydrase (CA) enzymes catalyze the interconversion of CO_2_ and bicarbonate. These enzymes play important roles in cellular metabolism, CO_2_ transport, ion transport, and internal pH regulation. Understanding the metabolic role of CAs in the chemolithoautotropic bacterium *Ralstonia eutropha* is important for the development of high performance fermentation processes based on the bacterium’s capability to fix carbon using the Calvin-Benson-Bassham (CBB) cycle. Analysis of the *R. eutropha* H16 genome sequence revealed the presence of four CA genes: *can, can2, caa* and *cag*. We evaluated the importance of each of the CAs in the metabolism of *R. eutropha* by examination of growth and enzyme activity in gene deletion, complementation, and overexpression strains. All four purified CAs were capable of performing the interconversion of CO_2_ and HCO_3_^–^, although the equilibrium towards the formation of CO_2_ or HCO_3_^–^ differs with each CA. Deletion of *can,* encoding a β-CA, affected the growth of *R. eutropha*; however the growth defect could be compensated by adding CO_2_ to the culture. Deletion of the *caa*, encoding an α-CA, had the strongest deleterious influence on cell growth. Strains with deletion or overexpression of *can2* or *cag* genes exhibited similar behavior to wild type under most of the conditions tested. In this work, Caa was studied in greater detail using microscopy and complementation experiments, which helped confirm its periplasmic localization and determine its importance for robust growth of *R. eutropha*. A hypothesis for the coordinated role of these four enzymes in the metabolism of *R. eutropha* is proposed.

## Introduction

Carbon dioxide, bicarbonate, carbonic acid, and carbonate are key metabolites in all living systems, and the equilibrium of these different forms in living cells is important for proper physiological functioning. Carbonic anhydrase (CA) (EC 4.2.1.1) catalyzes the interconversion between carbon dioxide and bicarbonate (CO_2_ + H_2_O ↔ HCO_3_^–^ + H^+^). The CA-catalyzed metabolic hydration/dehydration of CO_2_ and HCO_3_^–^ supports physiological functions in the cell, even though the uncatalyzed (enzyme-free) reaction proceeds at significant rates [1].

There are five main classes of CA enzyme found in nature (α, β, γ, δ and ζ). There are no significant sequence similarities observed between representatives of the different classes, implying that CA enzymes evolved convergently in diverse biological systems [2,3]. The α-CA enzymes are the most studied to date and are found in many different genera. They are monomeric, zinc-containing enzymes with multiple isozymes, which have different expression patterns and localizations [3-6]. In α-CA enzymes, Zn^2+^ is coordinated by three amino acids (usually histidines) and a water molecule. The role of Zn^2+^ is to facilitate the deprotonation of water for the formation of a nucleophilic hydroxide ion. This hydroxide ion can then initiate a nucleophilic attack on the carbonyl group of CO_2_ and convert it into bicarbonate. Specifically, the +2 charge of the zinc ion associates with the oxygen atom of water, supplying positive charge to the hydroxide ion, which is then able to attack the CO_2_[2]. All CA classes present differences in their secondary, tertiary and quaternary structures. Enzymes of the α class are monomeric, β-CA enzymes are usually oligomeric and contain 2 to 6 monomers, and γ-CA enzymes associate as homotrimers. Also, the active site ligands are potentially different among the CA classes, although β-CA and γ-CA enzymes are believed to function in a manner similar to α-CAs, in which both use the same zinc hydroxide mechanism [7].

*Ralstonia eutropha* (also *Cupriavidus necator*) is a Gram-negative facultative chemoautotrophic betaproteobacterium. It is well known for its ability to produce polyhydroxyalkanoate under high carbon but limited nitrogen or phosphorus conditions [8,9]. The capacity of *R. eutropha* to grow autotrophically using CO_2_ as the sole carbon source has been recently explored and studied for the production of alternative biofuels [10,11]. Assimilation of CO_2_ during autotrophic growth of *R. eutropha* proceeds by the Calvin-Benson-Bassham (CBB) cycle [12] and requires large amounts of energy to fuel the synthesis of cellular building blocks. Organisms must have a reliable and efficient system of controlling intracellular pH and CO_2_ concentrations in order to carry out carbon fixation [13]. Cyanobacteria evolved carboxysomes as an efficient mechanism to increase CO_2_ concentration and consequently its fixation efficiency [14,15], but *R. eutropha* lacks this system although it contains its two main enzymes, CA and RuBisCO [16]. Besides the key CBB cycle enzyme, RuBisCO, CA is of great importance for fine-tuning the concentration of CO_2_ in autotrophic metabolism.

Four putative CA genes were identified in the genome sequence of *R. eutropha* strain H16. Two CA genes are located on chromosome 1, and the others are on chromosome 2. The *can* (locus tag H16_A0169) and *can2* (locus tag H16_B2270) genes encode β-CA enzymes, the *caa* (locus tag H16_B2403) gene encodes a putative periplasmic α-CA, and the gene with locus tag H16_A1192 (hereafter known as *cag*) encodes a γ-like-CA/acetyltransferase [16]. The presence of CA genes of multiple classes in *R. eutropha* suggests that the gene products play major roles in CO_2_ transport and metabolism. Additionally, the diversity of CA gene products expressed in *R. eutropha* implies that the functions of these different enzymes could all be unique. Dobrinski et al. [17] examined four CA enzymes (α, β, γ and CsoSCA) from the deep sea proteobacterium *Thiomicrospira crunogena* and suggested different roles for each of the enzymes in relation to carbon fixation capabilities and survival mechanisms of the microorganism. Currently, the exact roles of all four *R. eutropha* CA enzymes are still largely unknown and the only one studied in depth to date is Can, which was identified as being essential for growth under atmospheric concentrations of CO_2_[1].

In the present study, we examined the activities of all four CA enzymes from *R. eutropha,* following heterologous expression and purification from *Escherichia coli*. We also assessed the effects of single and combinatorial CA gene deletions on cell physiology and fitness. The importance of Caa localization in the cell was further examined by overexpressing the enzyme with and without a periplasmic localization signal peptide in a Δ*caa* strain. Periplasmic localization was confirmed by detection of a fusion protein of Red Fluorescent Protein (RFP) and Caa using fluorescent microscopy.

## Materials and methods

### Chemicals, bacterial strains and plasmids

Chemicals were purchased from Sigma-Aldrich unless indicated otherwise. Experiments were performed with strains and plasmids listed in Table 1.

**Table 1:** Plasmids and bacterial strains used in this work with relevant genotype characteristics

**Strain or plasmid**	**Relevant characteristics**	**Reference or source**
*Ralstonia eutropha*		
H16	Wild-type gentamycin resistant (Gen^r^)	ATCC 17699
Re2061	H16 ∆*phaCAB* (Gen^r^)	[11]
Re2427	H16 ∆*can*	This study
Re2428	H16 ∆*caa*	This study
Re2430	H16 ∆*cag*	This study
Re2437	H16 ∆*can2*	This study
Re2436	H16 ∆*can*∆*can2*∆*caa*∆*cag*	This study
***Escherichia coli***		
DH10-*beta* competent cells	Strain suitable for high efficiency transformation	New England Biolabs
BL21(DE3)	Strain suitable for transformation and protein expression	New England Biolabs
S17-1	Conjugation strain for transfer of plasmids into *R. eutropha*	[18]
**Plasmids**		
pBBR1MCS-2	Broad-host-range cloning vector confers kanamycin resistance (Kan^r^)	[19]
pCan	pBBR1MCS-2 containing *can* gene (H16 A0169) (Kan^r^)	This work
pCan2	pBBR1MCS-2 containing *can2* gene (H16 B2270) (Kan^r^)	This work
pCaa	pBBR1MCS-2 containing *caa* gene (H16 B2403) (Kan^r^)	This work
pCaaB	pBBR1MCS-2 containing *caa* gene without the *N* terminal signaling peptide sequence (H16 B2403) (Kan^r^)	This work
pCag	pBBR1MCS-2 containing *cag* gene (H16 A1192) (Kan^r^)	This work
pETCan	pET14b containing *can* gene (H16 A0169) (Amp^r^)	This work
pETCan2	pET14b containing *can2* gene (H16 B2270) (Amp^r^)	This work
pETCaa	pET14b containing *caa* gene (H16 B2403) (Amp^r^)	This work
pETCag	pET14b containing *cag* gene (H16 A1192) (Amp^r^)	This work
pStrepCan	pET51b containing *can* gene (H16 A0169) (Amp^r^)	This work
pStrepCaa	pET51b containing *caa* gene (H16 B2403) (Amp^r^)	This work
pStrepCaaB	pET51b containing *caa* gene without the *N* terminal signaling peptide sequence (H16 B2403) (Amp^r^)	This work
pRARE	Overcoming the codon bias of *E. coli* for enhanced protein expression (Cam^r^)	[20]
pJV7	pJQ200Kan with *∆phaC1* inserted into *BamHI* restriction site, confers kanamycin resistance (Kan^r^)	[9]
pJV7 *∆can*	pJV7 with *∆phaC1*allele removed by *BamHI* digestion and replaced with *∆can* allele (Kan^r^)	This work
pJV7 *∆can2*	pJV7 with *∆phaC1*allele removed by *BamHI* digestion and replaced with *∆can2* allele (Kan^r^)	This work
pJV7 *∆caa*	pJV7 with *∆phaC1*allele removed by *BamHI* digestion and replaced with *∆caa* allele (Kan^r^)	This work
pJV7 *∆cag*	pJV7 with *∆phaC1*allele removed by *BamHI* digestion and replaced with *∆cag* allele (Kan^r^)	This work
pRFP	pBBR1MCS-2 containing *rfp* gene amplified from JBp000066 kindly offered by J. Mueller (JBEI) (Kan^r^)	This work
pCAA_RFP	pBBR1MCS-2 containing *caa* gene fused by a 6aa linker to the *rfp* gene (Kan^r^)	This work

### Growth media and cultivation conditions

*R. eutropha* strains were propagated in tryptic soy broth (TSB) (Becton Dickinson, Sparks, MD) or minimal medium [11] with fructose at a final concentration of 1% or 2% (w vol^-1^), or pyruvate, lactate, succinate, or formate, each at a final concentration of 0.2% (w vol^-1^) or (vol vol^-1^). All cultures were inoculated to an initial OD_600nm_ of 0.05. *E. coli* strains were grown in LB medium [21] at 37°C. For growth experiments in a CO_2_-rich environment, cultures were performed inside a CO_2_ incubator (Napco 6100 - Thermo Electron Corporation, Winchester, VA USA) with an atmosphere of 10% CO_2_ at 30°C, under 200 rpm agitation. Appropriate antibiotics were added to the growth media at the following concentrations: gentamicin, 10 μg mL^-1^; kanamycin, 200 μg mL^-1^ (for *R. eutropha*); kanamycin, 50 μg mL^-1^ (for *E. coli*); ampicillin, 100 μg mL^-1^; chloramphenicol, 34 μg mL^-1^.

Autotrophic cultures were prepared using minimal media without carbon source. For these cultures, a 250-mL Erlenmeyer flask containing 30 mL of culture is placed into a 15000 Vacu-Quik Jar System (Almore International, Inc., Portland, OR, USA). Agitation inside the flask was assured by a stir bar. Vacuum and nitrogen were applied alternatively and repeatedly three times to flush the chamber and remove all air. An approximate molar ratio of 8:1:1 (H_2_: O_2_: CO_2_) [22] gas mixture was then supplied to the jar. Any additional space in the jar was filled with 100% nitrogen.

### Plasmid and strain construction

Standard protocols were employed for DNA manipulation [23]. PCR amplification of DNA was performed using Phusion DNA Polymerase (New England Biolabs, Ipswich, MA, USA). Restriction enzymes and T4 DNA ligase used in this study were from New England Biolabs (Ipswich, MA, USA). The QIAquick Gel Extraction Kit (Qiagen, Valencia, CA, USA) was used for all gel purifications of DNA products and plasmid extractions were carried out using QIAprep Spin Miniprep Kit.

For the construction of overexpression and complementation plasmids, each of the four CA genes was cloned into pBBR1MCS-2 between restriction sites *KpnI* and *HindIII* (*can*, *can2* and *caa*) or *SalI* and *HindIII* (*cag*) to create the plasmids pCan, pCan2, pCaa and pCag respectively (Table 1). Each plasmid was transformed by electroporation into *E. coli* S17-1, which was then used as a donor strain for the conjugative transfer of plasmid into *R. eutropha* by a standard mating procedure [24]. For control experiments, *R. eutropha* was also transformed with the empty vector pBBR1MCS-2.

For the construction of plasmids for heterologous overexpression of CA in *E. coli*, followed by protein purification, CA genes were amplified and cloned as described above using the restriction sites with *NdeI* and *XhoI* (*can*, *can2* and *caa*) or *NdeI* and *BamHI* (*cag*) on both plasmids pET14b (*N*-terminal His-tag) and pET51b (*C*-terminal Strep2-tag) (Table 1). Each plasmid was then transformed into *E. coli* BL21 (DE3) harboring pRARE (Table 1).

For the assessment of the role of the predicted Caa signaling peptide and the effect of the correct localization of the enzyme on *R. eutropha* physiology, the entire annotated gene (*caa* -H16_B2403), with (*caa*) and without (*caaB*) the nucleotide sequence encoding the 23-aa *N*-terminal predicted signal-peptide were amplified and separately cloned into expression vectors (Table 1). The 23-aa *N*-terminal periplasmic signal peptide of Caa was predicted using SignalP 4.0 software [25].

RFP fusion plasmids were prepared by amplifying and inserting the *rfp* gene from the plasmid JBp000066 (kindly provided by J. Mueller and S. Singer – JBEI, Emeryville, CA, USA) at the 3′ end of the *caa* gene, with DNA sequence encoding a 6-glycine linker between the genes, into pBBR1MCS-2 using the Gibson assembly method [26] to produce pCaa_RFP. The *rfp* gene alone was expressed separately as a control (pRFP). Plasmids were introduced separately, as described above, into *R. eutropha* strain Re2061 (Table 1), which does not produce intracellular polyhydroxyalkanoate.

Plasmids for markerless deletion of CA genes were constructed according to Lu et al. [11]. Once the deletion strains Re2427 (H16 ∆*can*), Re2428 (H16 ∆*caa*), Re2430 (H16 ∆*cag*), and Re 2437(H16 ∆*can2*) were constructed, overexpression plasmids containing *can, can2, caa* or *cag* genes were introduced *in trans* to create Re2427/pCan, Re2428/pCaa, Re2430/pCag, Re2437/pCan2 and Re2428/pCaaB for complementation studies. A strain with quadruple CA gene deletions, Re2436 (H16∆*can*∆*can2*∆*caa*∆*cag*), was also constructed, and each CA enzyme, expressed *in trans*, was complemented back into the strain to create Re2436/pCan, Re2436/pCaa, Re2436/pCag and Re2436/pCan2. All oligonucleotide primers used for plasmid and strain constructions are listed in the Additional file 1: Table S1.

### Cell preparation and protein purification

*R. eutropha* CA genes were expressed in *E. coli* BL21(DE3)/pRARE using plasmids listed in Table 1. Cells were induced with IPTG (Isopropyl β-D-1-thiogalactopyranoside, 0.6 mM final concentration) at a culture OD_600_ of 0.4-0.6. Cells were harvested 8 h post-induction by centrifugation and stored at -80°C prior to CA purification and activity assay. Purification of Strep2-tagged proteins was performed using a gravity flow Strep-Tactin® Superflow® high capacity column (IBA, Göttingen, Germany) following the manufacturer’s instructions. His-tagged proteins were purified using a 5-mL HisTrap® FF column (Amersham Bioscience, Uppsala, Sweden) operated by a low pressure liquid chromatography system (BioRad, Hercules, California, USA). Fractions containing purified CA were detected on a 12% SDS-PAGE gel [23], concentrated using Amicon Ultra-15 Centrifugal Filter Units (EMD Millipore Corporation, Billerica, MA, USA), and dialyzed overnight using a Slide-A-Lyzer Dialysis Cassette (Thermo Scientific, Asheville, NC, USA) in Tris–HCl (100 mM pH 7) at 4°C.

### Determination of CA activity

The CA activity assays were based on methods from Sundaram et al. [27] and Fasseas et al. [4] with some modifications. CA activity was measured by comparing the rapid change of pH in the presence of CA to the slower pH change in the absence of CA. The assays were performed using a stopped-flow device (Applied Photophysics Ltd, Leatherhead, United Kingdom) connected to a spectrophotometer (Agilent 8453 UV-Visible Kinetic Mode). Purified CA enzymes were diluted in assay buffer (50 mM Na_2_SO_4_, 50 mM HEPES, 50 mM MgSO_4_, 0.004% (w v^-1^) Phenol red, pH 8 for assays using CO_2_ as a substrate or pH 6 for assays using KHCO_3_ as a substrate. All reagents were kept on ice while the spectrophotometer and stopped-flow apparatus were kept at 1°C ± 1°C using a recirculating water bath. The substrates used for the activity assays were KHCO_3_ (50 mM) and saturated CO_2_ (dry ice in assay buffer for 30 min). The final volume of the assay reaction was 400 μL and the absorbance A_557nm_ was measured for 60 sec. Control assays were carried out in the absence of enzymes.

Enzyme Units (EU) were calculated as illustrated below by comparing the time of pH change in the presence of enzyme to reaction without enzyme [27]. Each additional EU speeds the catalytic activity of the enzyme by two fold. The assay was validated using commercially available purified CA from bovine erythrocytes (Sigma-Aldrich C9207). Protein concentration (purified or cell extract) was determined by standard Bradford assay [28].

$$\mathrm{EU}=\frac{\text{Time}\;\mathrm{of}\;\text{uncatalized}\;‒\text{Time}\;\mathrm{of}\;\text{catallized}\;\text{reaction}\;(s)}{\text{Time}\;\mathrm{of}\;\text{catalized}\;\text{reaction}\;(s)}\times \frac{1}{\text{Total}\;\text{protein}\;\mathrm{in}\;\text{assay}\;\left(\mathrm{mg}\right)}$$

Adapted from [27].

### Microscopy

Slides of Re2061/pCaa_RFP or Re2061/pRFP were prepared using 10 μL of overnight TSB cultures placed onto Poly-*L*-Lysine-coated slides. Cells were observed under 100× magnification using a Nikon Labophot-2 microscope with phase-contrast and fluorescence attachments. Images were acquired with a SPOT cooled color digital camera (Diagnostic Instruments, Inc.) and prepared using Adobe Photoshop version 5.0.

### Polymer quantification

Polyhydroxybutyrate (PHB) content was determined as described previously [29,30].

## Results

### Characterization of CA enzymes

**Figure 1:**
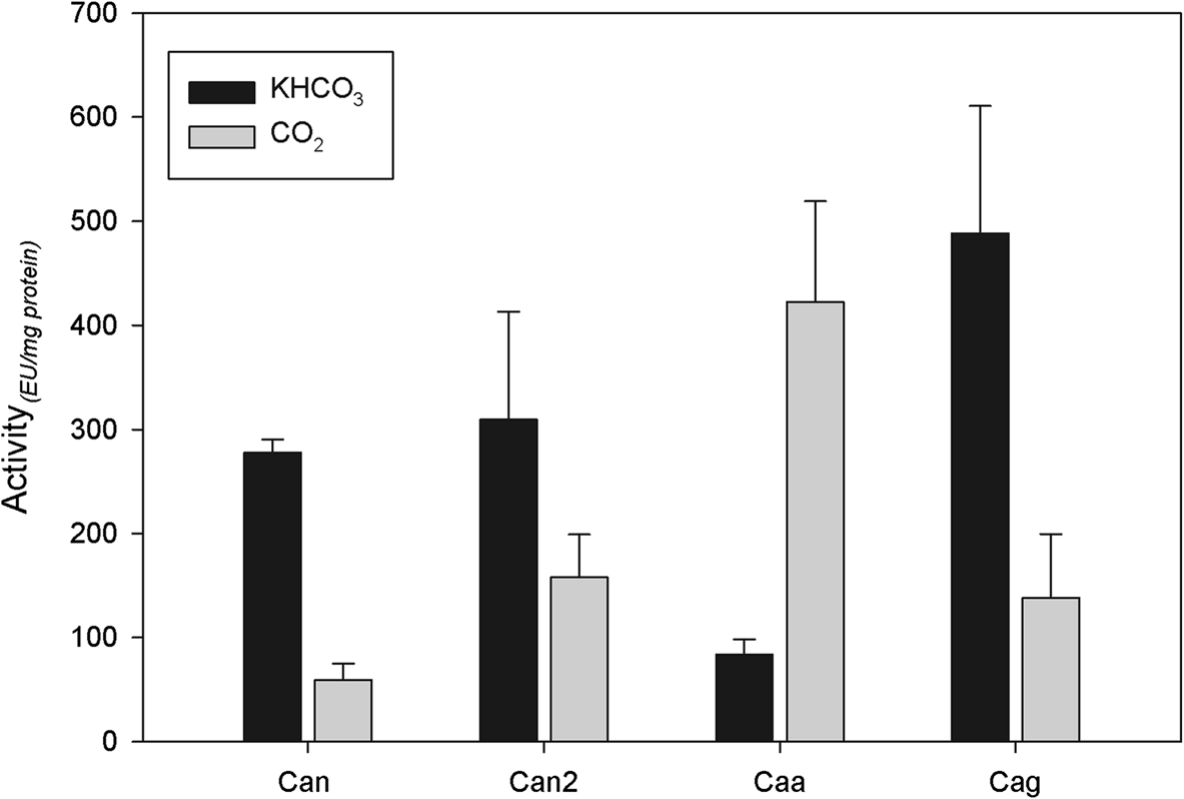
**Specific activity of purified Carbonic Anhydrases.** Specific activity of purified, His-tagged carbonic anhydrase enzymes. Substrates used for this assay were bicarbonate (50 mM, dark bars) and CO_2_ (saturated solution, grey bars). CA activity was measured by comparing the rapid change of pH in the presence of CA to the slower pH change in the absence of CA. The pH change was monitored using phenol red and the absorbance A_557nm_ was measured for 60 sec. Average values from three experiments were plotted with error bars representing the standard deviation.

All four carbonic anhydrase enzymes were heterologously overexpressed in recombinant *E. coli,* purified, and examined in this work. Each CA was able to catalyze the interconversion reaction between CO_2_ and bicarbonate. Additionally, each CA exhibited different specific activity and substrate preference towards CO_2_ and bicarbonate. Caa presented a higher activity using CO_2_ as the substrate, compared to the activity of Can, Can2 or Cag, which all preferred bicarbonate (Figure 1). The specific activity of Caa towards CO_2_ as a substrate (422.2 ± 97.1 EU mg^-1^) was higher than the activity measured for the other CAs (Can 59.5 ± 15.3 EU mg^-1^; Can2 157.9 ± 4 EU mg^-1^ and Cag 138.2 ± 61.3 EU mg^-1^). On the other hand, the specific activities of both Can and Can2 were similar with bicarbonate as a substrate (277.9 ± 12.6 EU mg^-1^ and 309.4 ± 103.1 EU mg^-1^ respectively). Cag presented the highest activity using bicarbonate as a carbon source (488.5 ±122.1 EU mg^-1^). All CAs were active towards CO_2_ and bicarbonate, however only Caa had a distinct preference for CO_2_ as a substrate while the other three CAs all preferred bicarbonate. The tests for validation of the assay were done using a commercial CA and the values obtained were 130.7 ± 20.1 EU mg^-1^ using bicarbonate as a substrate and 168.8 ± 5.9 EU mg^-1^ using CO_2_ as a substrate. Comparative CO_2_ hydration values reported for CAs of different microorganisms can be found on Additional file 1: Table S2.

### Growth characterization of overexpression strains

**Figure 2:**
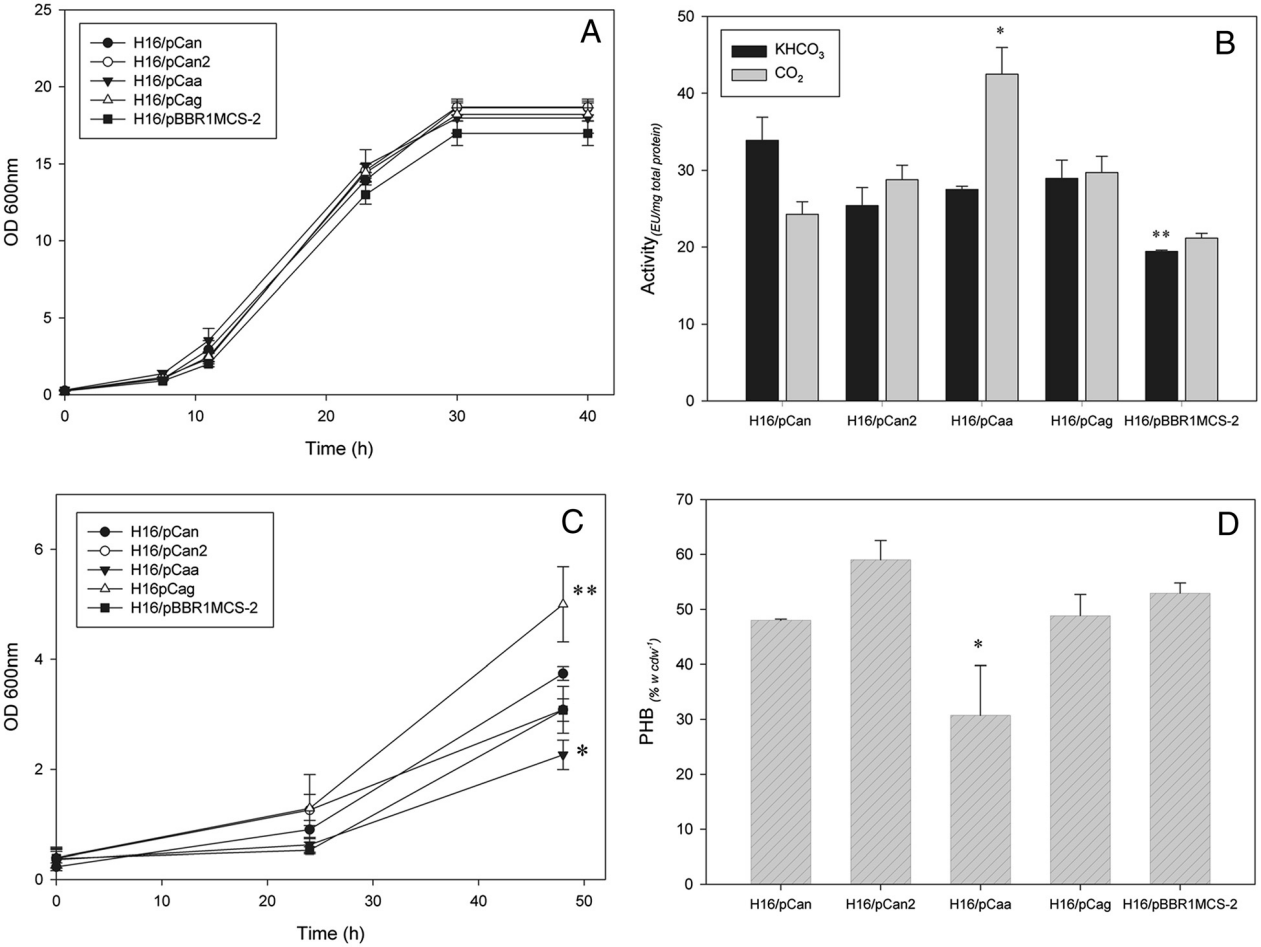
**Growth, CA activity and PHB production of overexpression strains. A)** Growth of CA overexpression strains H16/pCan, H16/pCan2, H16/pCaa and H16/pCag, compared to the control strain (H16/pBBR1MCS-2) with 2% (w vol^-1^) fructose as the main carbon source. **B)** Carbonic anhydrase activity of overexpression strains after 24 h of growth in minimal medium with 2% (w vol^-1^) fructose. H16/pBBR1MCS-2 was used as a control. The Tukey’s test was used as statistic test to compare all the strains (*) represents significant difference in activity using CO_2_ as a substrate. (**) represents significant difference in activity using bicarbonate, P < 0.01. **C)** Growth of *R. eutropha* strains H16/pCan, H16/pCan2, H16/pCaa and H16/pCag and control strain H16/pBBR1MCS-2 under autotrophic conditions. (*) represents significant difference in final OD_600nm_ P < 0.01. (**) represents significant difference in final OD_600nm,_ P < 0.05. **D)** PHB production in 48 h by the overexpression and control strains and under autotrophic conditions. (*) represents significant difference in PHB production per cell dry weight (CDW) (w w^-1^), P < 0.01. Values represented were averages from three replicates with standard deviation represented by error bars.

The growth of CA overexpression strains H16/pCan, H16/pCan2, H16/pCaa and H16/pCag were tested in minimal medium with 2% (w vol^-1^) fructose in the presence of ambient CO_2_ concentrations (air) and under autotrophic condition (H_2_:CO_2_:O_2_). Using fructose as the carbon source, all strains exhibited similar growth behavior (Figure 2A)

CA activity of cell extracts of *R. eutropha* strains grown on fructose was determined. As shown in Figure 2B, the CA-overexpression strains exhibited higher activity than the control using both substrates. Notably high CA activity was observed in H16/pCaa using CO_2_ as a substrate. Using bicarbonate as a substrate, all the strains presented significantly higher activity than the control (P < 0.01). However, each CA overexpression strain exhibited no significant difference in activity using bicarbonate as the substrate. These results indicate the preference of Caa for CO_2_ as a substrate, which is in accordance with activity results using purified Caa (Figure 1). The PHB content of the CA overexpression and control strains was also assessed, and it was observed that all strains grown using fructose as the main carbon source produced similar intracellular amounts of PHB (data not shown).

Under autotrophic conditions, significant differences in growth were observed between the different strains. Growth of H16/pCaa was significantly lower than all the other strains (P < 0.01). H16/pCag grew to a greater extent compared to the others (P < 0.05), while H16/pCan and H16pCan2 presented no significant growth difference compared to H16/pBBR1MCS-2 (Figure 2C). These strains were tested for PHB production after 48 h of autotrophic growth (Figure 2D). H16/pCaa produced the lowest amount of PHB per CDW, compared to the other CA overexpression and control strains.

### *Growth characteristics of the* CA *deletion strains*

**Figure 3:**
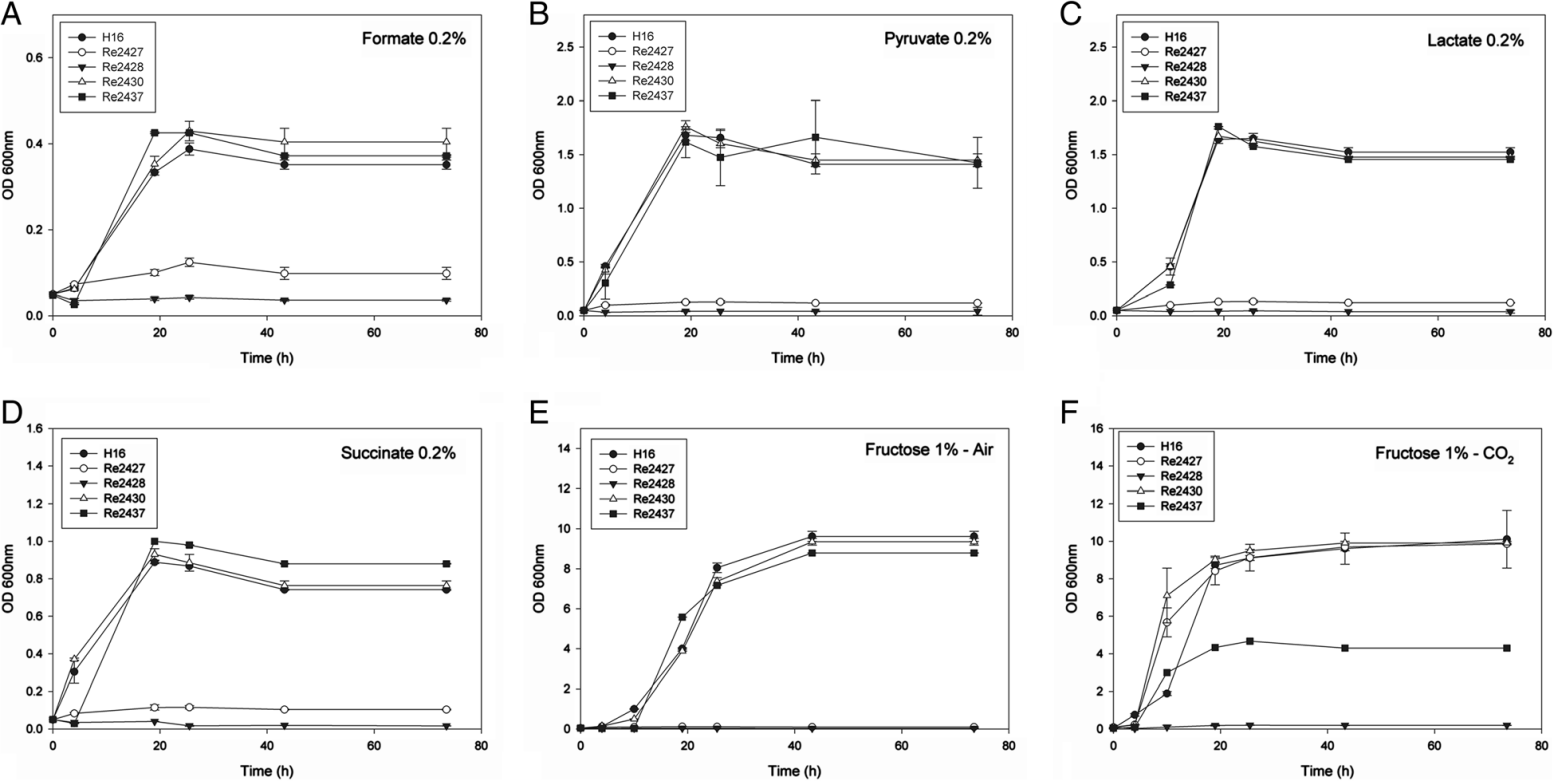
**Growth of deletion strains in different carbon sources. A)** Growth of *R. eutropha* H16 (wild type - *●*) and CA deletion strains Re2427 (H16∆*can* - ○), Re2428 (H16∆*caa* -▼), Re2430 (H16∆*cag* - ∆) and Re2437 (H16∆*can2 -* ■) on different carbon sources: 0.2% (vol vol^-1^) formate (A), 0.2% (w vol^-1^) pyruvate **(B)**, 0.2% (vol vol^-1^) lactate **(C)** and 0.2% (w vol^-1^) succinate **(D)**, 1% (w vol^-1^) fructose **(E)**, and 1% (w vol^-1^) fructose supplemented with 10% CO_2_**(F)**. Values represented averages from three replicates with standard deviation as error bars.

The deletion strains (Re2427, Re2428, Re2430 and Re2437) were cultivated in minimal medium supplemented with various carbon sources (fructose, formate, pyruvate, succinate, or lactate) in the presence of air or air supplemented with 10% CO_2_. Strain Re2428 (H16∆*caa*) was unable to grow under all conditions tested, except in fructose with excess CO_2,_ where it grew slightly to a final OD_600nm_ of 0.2 (Figure 3F). Growth of strain Re2427 (H16∆*can*) was completely recovered with the addition of CO_2_ to the fructose culture (Figure 3F), although this strain grew poorly under other nutrient-supplemented conditions (Figure 3A-E). Strain Re2437 (H16∆*can2*) grew no differently than the wild type, except in the presence of fructose supplemented with CO_2_, in which the growth output dropped by nearly 60%. Re2437 cells presented different morphology under light microscopy without CO_2_ supplement. These cells were longer than the wild type cells under such condition, (Additional file 1: Figure S2) which can be a sign of stress that was not detected during absorbance measurements and could be caused by a pH imbalance during growth. Additional file 1: Figure S2 shows light microscopy images of the deletion strains. In the presence of increased CO_2_ concentrations, increasing stress could be detected by an observed decrease in absorbance (Figure 3F). This suggests the importance of Can2 in maintaining the cellular pH, because the addition of CO_2_ to the media decreases the pH in the media, affecting consequently the cytoplasmic pH. To test this, strain Re2437 was cultivated in media under different pH values (5.5, 7.0 and 8.5). The growth of Re2437 was less affected by this pH change as compared to wild type. Growth of *R. eutropha* H16 and Re2427 with different initial pH values can be seen in Additional file 1: Figure S3. The growth of Re2430 (H16∆*cag*) was similar to wild type under all conditions tested.

### Investigation of the phenotypic effects of individual CA enzymes

The quadruple mutant, Re2436 (H16 ∆*can* ∆*can2* ∆*caa* ∆*cag*), had each of the four CA-encoding genes inserted *in trans* to create Re2436/pCan, Re2436/pCaa, Re2436/pCag, and Re2436/pCan2 (see Materials and methods), in order to demonstrate the isolated effect of each CA on *R. eutropha* metabolism.

**Figure 4:**
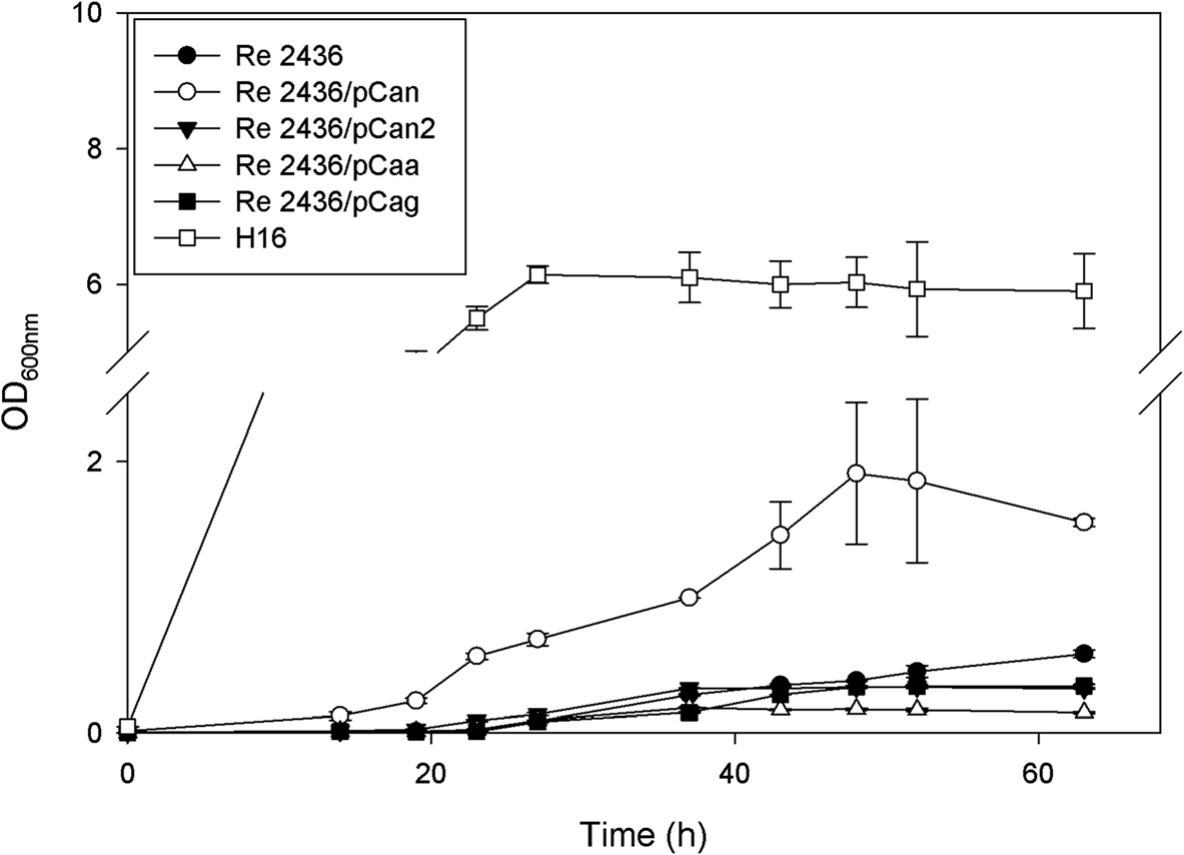
**Growth of the quadruple deletion mutant on TSB + 10% CO**_**2**_**.** Cultures of *R. eutropha* H16 (□), *R. eutropha* CA quadruple deletion mutant Re2436 (H16 ∆*can* ∆*can2* ∆*caa* ∆*cag* - ●) and the complemented strains, Re2436/pCan (○), Re2436/pCan2 (▼), Re2436/Caa (∆) and Re2436/pCag (■) in TSB media in the presence of 10% CO_2_. Values represent average from three replicates with standard deviation as error bars.

As expected, none of the strains completely recovered the phenotype when growing on minimal media with fructose as a carbon source in air or CO_2_-enriched environment (data not shown). In ambient air condition, no significant difference was observed with growth of all four strains compared to wild type (data not shown). On the other hand, with the addition of 10% CO_2_, Re2436/pCan exhibited a higher growth rate when compared to Re2436 and the other strains (Figure 4).

The CA single deletion strains were transformed with the respective plasmid to compliment the CA deletion to obtain Re2427/pCan, Re2428/pCaa, Re2430/pCag and Re2437/pCan2. When tested on minimal media with fructose 2% (w vol^-1^) the strains Re2427/pCan, Re2430/pCag and Re2437/pCan2 exhibited growth comparable to wild type. Growth of complemented deletion strains can be seen in Additional file 1: Figure S1. The only exception was Re2428/pCaa, which was unable to grow to an extent comparable with the wild type under any condition tested and was therefore the subject of closer examination in this study.

### A closer look at Caa

Examination of the α-periplasmic enzyme, Caa, yielded very interesting results during this study. Besides being the only α-carbonic anhydrase identified in the genome sequence of *R. eutropha* strain H16, it is described as a “putative periplasmic enzyme.” In this study, the purified Caa is the only *R. eutropha* CA capable of performing the interconversion of CO_2_ and HCO_3_^–^ with equilibrium lying towards the formation of HCO_3_^–^ (Figure 1). The results of Caa overexpression in *R. eutropha* H16 grown in autotrophic culture indicated that the overexpression of Caa in the wild type strain had a negative influence on growth (Figure 2C) and PHB production (Figure 2D). Moreover, in any medium tested, the *caa* deletion strain, Re2428, was unable to recover growth to wild type levels (Figure 3).

**Figure 5:**
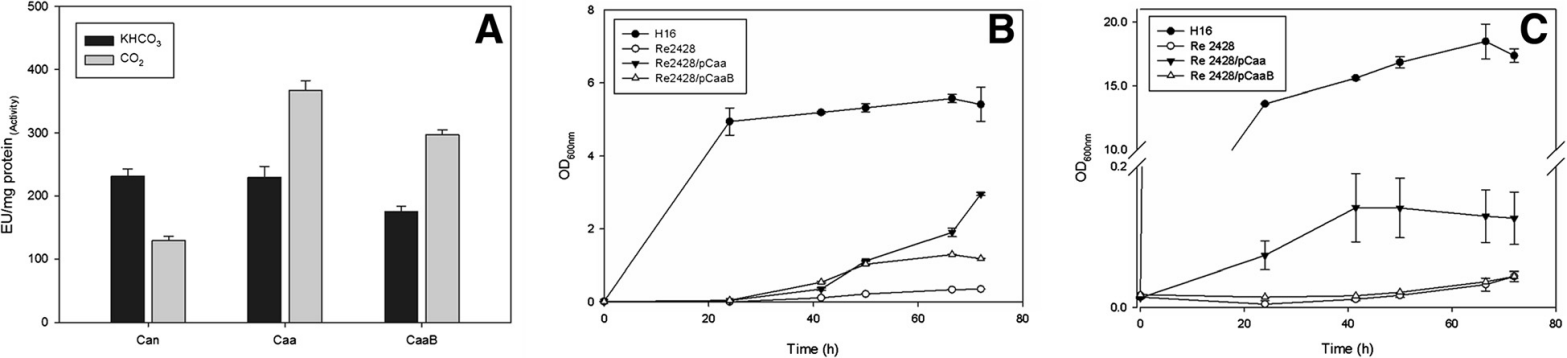
**Specific activity of Caa. Growth of complementation strain in TSB and minimal media with fructose. A)** Specific activity of purified Strep2-tagged CA enzymes. Substrates used for this assay were bicarbonate (50 mM, dark bars) and CO_2_ (saturated solution, grey bars). CA activity was measured by comparing the rapid change of pH in the presence of CA to the slower pH change in the absence of CA. The pH change was monitored using phenol red and the absorbance A_557nm_ was measured for 60 sec. Average values from three experiments were plotted with error bars representing the standard deviation. **B)** Cultures of *R. eutropha* wild type (H16 - ●), Re2428 (○), Re2428/pCaa (▼), and Re2428pCaaB (∆) in TSB media. **C)** Growth in minimal media with 2% fructose (w vol^-1^). Values represent average from three replicates with standard deviation as error bars.

In order to study the importance of Caa cellular localization on the phenotype recovery of Re2428, *caa* was cloned both as annotated and without the *N*-terminal signaling peptide, which hypothetically would prevent it from being targeted to the periplasm of *R. eutropha*. Purified, Strep2-tagged Caa enzyme without the signaling peptide (CaaB) exhibited similar activity values when compared to the purified, Strep-tagged Caa enzyme containing the signaling peptide. Purified Can was used as a control for CA activity assay (Figure 5A).

The *caa* and *caaB* genes were reintroduced *in trans* into Re2428 using plasmids pCaa and pCaaB (Table 1), in an attempt to recover *R. eutropha* growth in air and to determine the effect of Caa localization on cell growth. *R. eutropha* wild type, Re2428, and complemented strains (Re2428/pCaa and Re2428/pCaaB) were cultivated in TSB and minimal media. The results presented in Figure 5B and C show that Re2428/pCaa and Re2428/CaaB grew poorly under the conditions tested when compared to the wild type strain. In TSB media, after 72 h of culture, the presence of Caa or CaaB in Re2428 resulted in OD_600nm_ of 2.96 ± 0.039 and 1.18 ± 0.009 respectively, both of which were much lower than growth of the wild type (OD_600nm_ = 4.95 ± 0.35). In complementation strains, the localization of Caa in the cell during growth on TSB media did not present a significant difference (Figure 5B). In minimal media, however, a partial recovery of growth was observed only for Re2428/Caa (Figure 5C). These results indicate that overexpression of *caa* in Re2428 was incapable of recovering growth to wild type rates in either rich or minimal medium, even though CA activity was detected in cell extracts of Re2428/pCaa and Re2424/pCaaB (data not shown). The periplasmic localization of Caa, under the conditions tested, was only observed to affect growth of the cells to a significant extent when cultivated in minimal media.

### Localization of Caa

In order to provide evidence for the periplasmic localization of Caa in *R. eutropha*, RFP was fused to the C-terminus of Caa to allow visualization of the localization in the cell.

RFP-tagged Caa and RFP alone (control) were expressed separately in the *R. eutropha* strain Re2061, which is unable to produce intracellular polyhydroxyalkanoate that may disturb the imaging. As shown in the fluorescent microscopy images presented in Additional file 1: Figure S4A, the fluorescence was concentrated near the outer perimeter of the cells with RFP-tagged Caa, as opposed to the RFP-only control, where fluorescence was diffused throughout the cytosol (Additional file 1: Figure S4B). This suggests that Caa enzymes are located near the outer perimeter of the cell, likely the periplasm.

## Discussion

In this study, we evaluated four putative CA genes found in *R. eutropha* genome. The *can* and *can2* genes encode β-CA enzymes, the *caa* gene encodes a putative periplasmic α-CA, and the *cag* gene encodes a γ-like-CA/acetyltransferase. We demonstrated the ability of all four CA enzymes to catalyze the interconversion of CO_2_ and bicarbonate, as purified enzymes and also when overexpressed in *R. eutropha*. We elucidated some of the complexities of CO_2_ metabolism in *R. eutropha* and the evolutionary importance for the bacteria to retain four separate CA genes, which apparently present similar activities in the cell, but which have unique roles within the machinery for controlling its CO_2_ metabolism. In short, the presence of one enzyme does not exclude the importance of the others.

Previous microarray data produced in our laboratory demonstrated different expression behavior of the four CA genes when *R. eutropha* was growing on fructose or trioleate in the presence of high or low concentrations of nitrogen [31]. The *can* gene exhibited an expression decrease of 1.7 fold (p = 0.0048) when cells entered into nitrogen depletion, *i.e.* the PHB production stage. On the other hand, *cag* exhibited an increase in expression of 3.5 fold (p = 0.011) during the same growth condition. When changing the carbon sources from triolate to fructose, *caa* exhibited an expression decrease of 3.0 fold (p = 0.001). All of these previous observations support the idea that the four enzymes are not responsible for the same reactions in the cell, although Kusian et al. [1] proposed that, out of the four CA enzymes described in *R. eutropha,* only *can* plays an important role on CO_2_ metabolism. Supporting our findings of independent roles of CAs in *R. eutropha*, [32-34] also reported and discussed the separate roles of different CAs expressed in the same organism. Dobrinski et al. [17] described four different CAs expressed at different levels depending on the physiological state of the microorganism in *T. crunogena* Among other results, the observed differences in expression levels indicate a specific role of each CA enzyme in carbon fixation, pH homeostasis or other physiological activities not yet elucidated.

Overexpression of the CA enzymes separately in the wild type strain resulted in no observable difference in growth (Figure 2A) or PHB production (data not shown), but higher CA activities were detected in the cell extracts when compared to the wild type harboring the empty vector (Figure 2B). Examination of the purified CA enzymes (Figure 1), and CA overexpression strains (Figure 2), showed that Caa exhibited greater activity using CO_2_ as a carbon source. Under autotrophic growth conditions, when CO_2_ is used as the sole carbon source, the growth and PHB accumulation of the CA overexpression strains presented significant differences when compared to each other and to the wild type strain (Figure 2C and D). One reason for the low growth and PHB production of the overexpression strains could be due to the altered regulation of gene expression.

The phenotype recovery effect of the overexpression of Caa in Re2428 (H16Δ*caa*) is more distinct when cells are grown in minimal media (Figure 5C), because in this environment, bicarbonate is required to synthesize essential amino acids and nucleotides, which are not supplied in the media [33].

We have identified and characterized an α-periplasmic CA, Caa, and demonstrated the importance of Caa for the transport of CO_2_ and the supply of bicarbonate to *R. eutropha* cells during growth. Caa deletion, imbalanced expression, and mislocalization all disabled and adversely affected cell growth, presumably by severely impairing *R. eutropha* metabolism.

Several essential metabolic pathways require either CO_2_ or bicarbonate as a substrate. Bicarbonate is the substrate of several important enzymes of central metabolism, such as phosphoenolpyruvate carboxylase, pyruvate carboxylase, acetyl-CoA carboxylase and methylcrotonyl CA, among others [3]. Calculated by Merlin et al. [33], the spontaneous diffusion of CO_2_ in air and conversion to bicarbonate inside the cell are not sufficient for the metabolic needs of a bacterial cell. In *R. eutropha* metabolism, bicarbonate is required for the elimination of a long lag phase during autotrophic growth [35], which is considered to be a negative feature for the use of this microorganism at industrial scale. CAII, a human α-CA, is known to play an important role in the transportation of CO_2_ and supplying bicarbonate to mammalian cells [36] and in *T. crunogena*. Dobrinski et al. [17] also propose that an α-CA would be converting CO_2_ to bicarbonate in the cells. In *R. eutropha*, Caa could be responsible for this same mechanism, since the equilibrium of Caa-catalyzed reaction lies towards the hydration of CO_2_ to bicarbonate (Figure 1). Can, alternatively, could be responsible for the supplementation of CO_2_. These hypotheses were supported by the deletion of *caa* or *can* from the genome of *R. eutropha*. The lack of *can* resulted in a phenotype in which cells were dependent on external CO_2_ supplementation in order to grow [1], this study. Without *caa* (i.e., strain Re2428), *R. eutropha* presented a similar phenotype as the *can* deletion strain, but the activities and substrate specificities of these two CA enzymes are remarkably different. Moreover, the growth phenotype of a *caa* deletion could not be compensated by the addition of CO_2_ to the culture. These differences imply disparate physiological roles of both of these CA enzymes in the growth and maintenance of *R. eutropha*.

Additionally, both Caa and Can could not replace the roles of one another in *R. eutropha*, because a single deletion of either *can* or *caa* resulted in no growth under ambient CO_2_ conditions [1], this study. Although *in vitro* activity suggested that the enzymes could have complementary activities (Figure 1). Overall, we conclude in this study that Caa is unable to act as a replacement for Can, and vice versa. This conclusion is supported by the physical separation of both CAs in the cell, in which Caa is located in the periplasm and Can is believed to be in the cytosol.

The attempt to recover fully the phenotype of Re2428 by overexpressing a plasmid-borne *caa* gene was unsuccessful, as the overall growth yields of the complementary strains Re2428/pCaa and Re2428/pCaaB were much lower than that of the wild type, even when both versions of the enzyme, after purification, exhibited high activity (Figure 5A). This could be the result of an expression level imbalance of the plasmid-borne *caa* compared to that of wild type. Interestingly, the strain Re2428/pCaaB was unable to grow at the same rate as Re2428/pCaa in minimal media (Figure 5C), although both complemented strains grew similarly in TSB medium (Figure 5B). The growth difference could be a result of the differential localization of the two versions of Caa in the cell. The lack of a signaling peptide may have prevented the periplasmic positioning of Caa in the cell, and thus resulted in a lower bacterial growth. This effect can be more clearly observed in minimal media (Figure 5C). The results suggest that Caa is a periplasmic CA and its correct location in the cell is essential for the growth of *R. eutropha*. Overall, reintroduction of *caa* or *caaB* in Re2428 was able to recover only partial growth.

The isolated effect of the CAs in the genome could be observed after complementing the quadruple mutant, Re2436 (H16Δ*can*Δ*caa*Δ*can2*Δ*cag*), with each separate enzyme expressed from a plasmid (Figure 4). In a background with no CA enzymes expressed, none of the individual CAs expressed *in trans* could fully recover the growth phenotype under all conditions tested. However, strain Re2436/pCan, expressing the Can enzyme, when grown in minimal media with fructose as the main carbon source could partially recover the growth phenotype (Figure 4). This reinforces the importance of having all four CAs in a complex system in order for carbon metabolism and cell growth to function properly.

**Figure 6:**
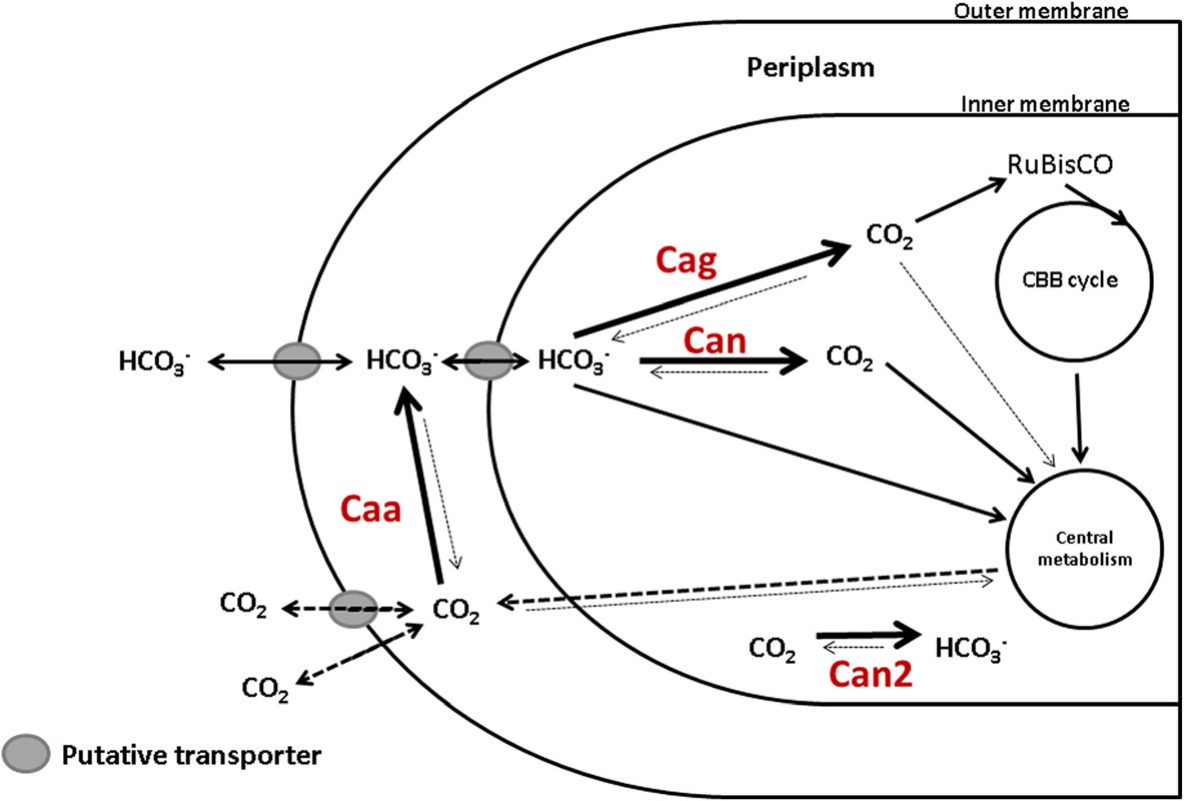
**Working CA roles on *R. eutropha *CO**_**2 **_**metabolism.** Schematic depiction of the role of CAs in *R. eutropha* metabolism. Based on experiments performed in this study, the role of Caa in *R. eutropha* is to convert the diffused CO_2_ inside the periplasm into bicarbonate to supply cellular metabolism. Can, and the less well understood Cag, would also be responsible for the supplementation of CO_2_ to RuBisCO. Can2, could play a role on the pH maintenance in the cell (see Additional file 1: Figure S3 which shows growth of *R. eutropha* H16 and Re2427 with different initial pH values). CBB cycle = Calvin-Benson-Bassham Cycle; RuBisCO = Ribulose-1,5-bisphosphate carboxylase oxygenase.

The localization of Caa in the periplasm of the cell is crucial for the conversion of CO_2_, which is passively diffused though the membrane, to dissolved inorganic carbon (bicarbonate). The conversion of CO_2_ to bicarbonate is important for the transportation of CO_2_ into the cell, as CO_2_ is extremely insoluble in aqueous solution and frequently diffuses in and out of the cell. Bicarbonate, on the other hand is negatively charged and highly soluble in aqueous solution, but poorly soluble in lipids. Transport of bicarbonate across the cell membrane must be assisted by CA [3,5]. Figure 6 depicts a diagram representing the role of CAs in *R. eutropha* cells. Can is likely the enzyme responsible for supporting CO_2_ fixation reaction by providing CO_2_ to RuBisCO in the cytosol. The same reaction can potentially be performed by Cag, but its role is elusive and thus merits further investigation. Caa is responsible for sequestering the CO_2_ diffused into the cell and converting it to bicarbonate for cellular metabolism, while Can2 has a possible role in pH homeostasis. The current work provides a springboard to understanding the differential roles of each CA enzyme in the physiology and CO_2_ homeostasis of *R. eutropha*. The exact interplay of each of these enzymes in the cell still continues to be an active area of study.

## Competing interest

The authors declare that there is no conflict of interest.

## Supplementary Material

Additional file 1: Figure S1 Growth of complemented deletion strains. **Figure S2.** Light microscopy of deletion strains. **Figure S3.** Growth of H16 and Re2427 with different initial pH values. **Figure S4.** Fluorescent microscopy of Caa_RFP fusion protein expressed in Re2061. **Table S1.** Oligonucleotide primers used in this study [37-43]. Click here for file
