# Supplementary material for: Insights into bacterial CO2 metabolism revealed by the characterization of four carbonic anhydrases in Ralstonia eutropha H16
Source: AMB Express. 2014 Jan 10;4:2. doi: 10.1186/2191-0855-4-2 (PMC3904209; doi:10.1186/2191-0855-4-2)
Supplement: Additional file 1: Figure S1 — Growth of complemented deletion strains. Figure S2. Light microscopy of deletion strains. Figure S3. Growth of H16 and Re2427 with different initial pH values. Figure S4. Fluorescent microscopy of Caa_RFP fusion protein expressed in Re2061. Table S1. Oligonucleotide primers used in this study [37-43]. [file 2191-0855-4-2-S1.docx]

Additional File 1

**
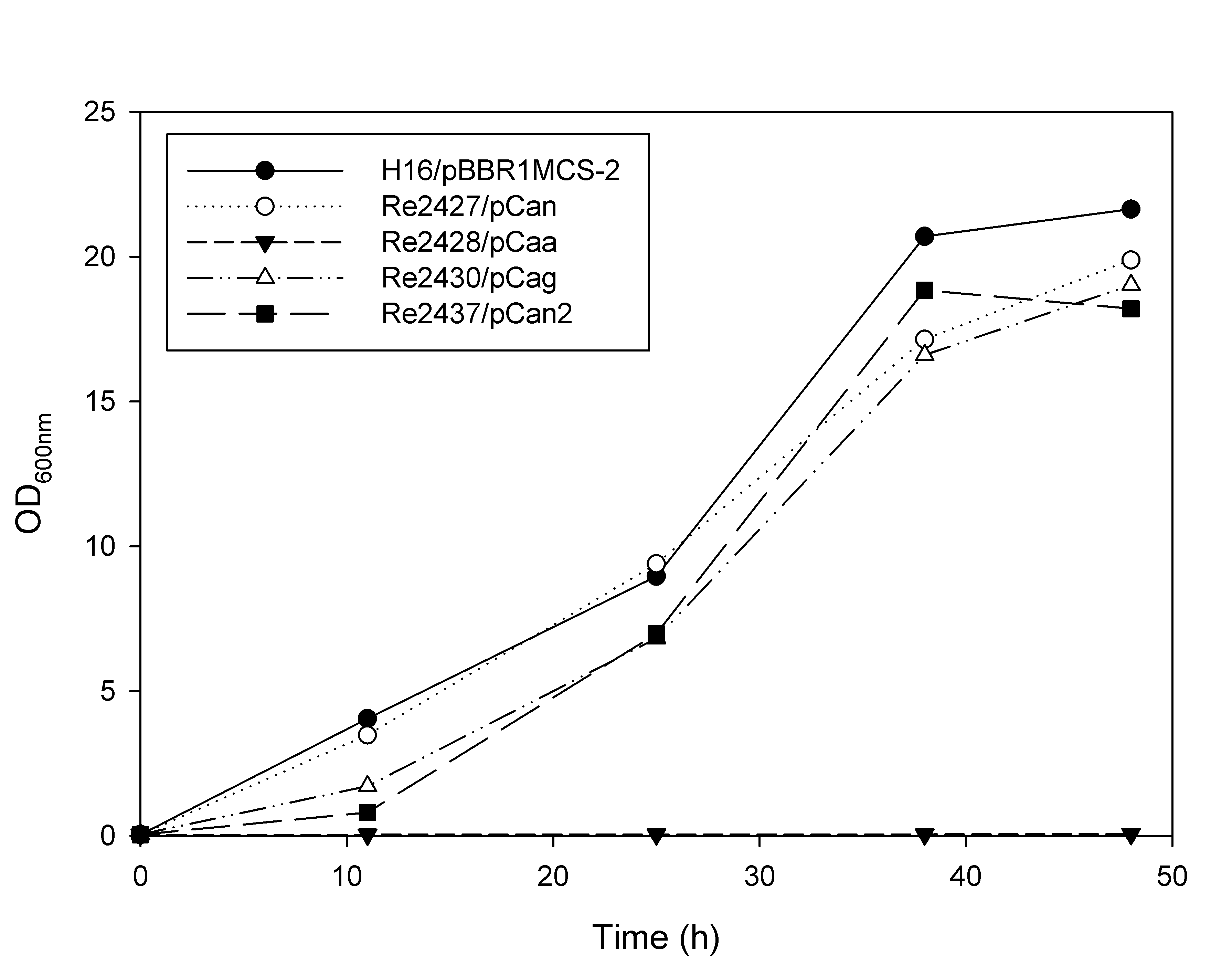
**

**Additional File 1 Figure S1 Growth of complemented deletion strains.**

Growth of the CA gene deletion strains, complemented with their respective plasmid-borne genes, in minimal media containing 2% (w v^-1^) fructose. The growth of all the mutants except Re2428/pCaa was completely recovered by the overexpression of each corresponding CA.


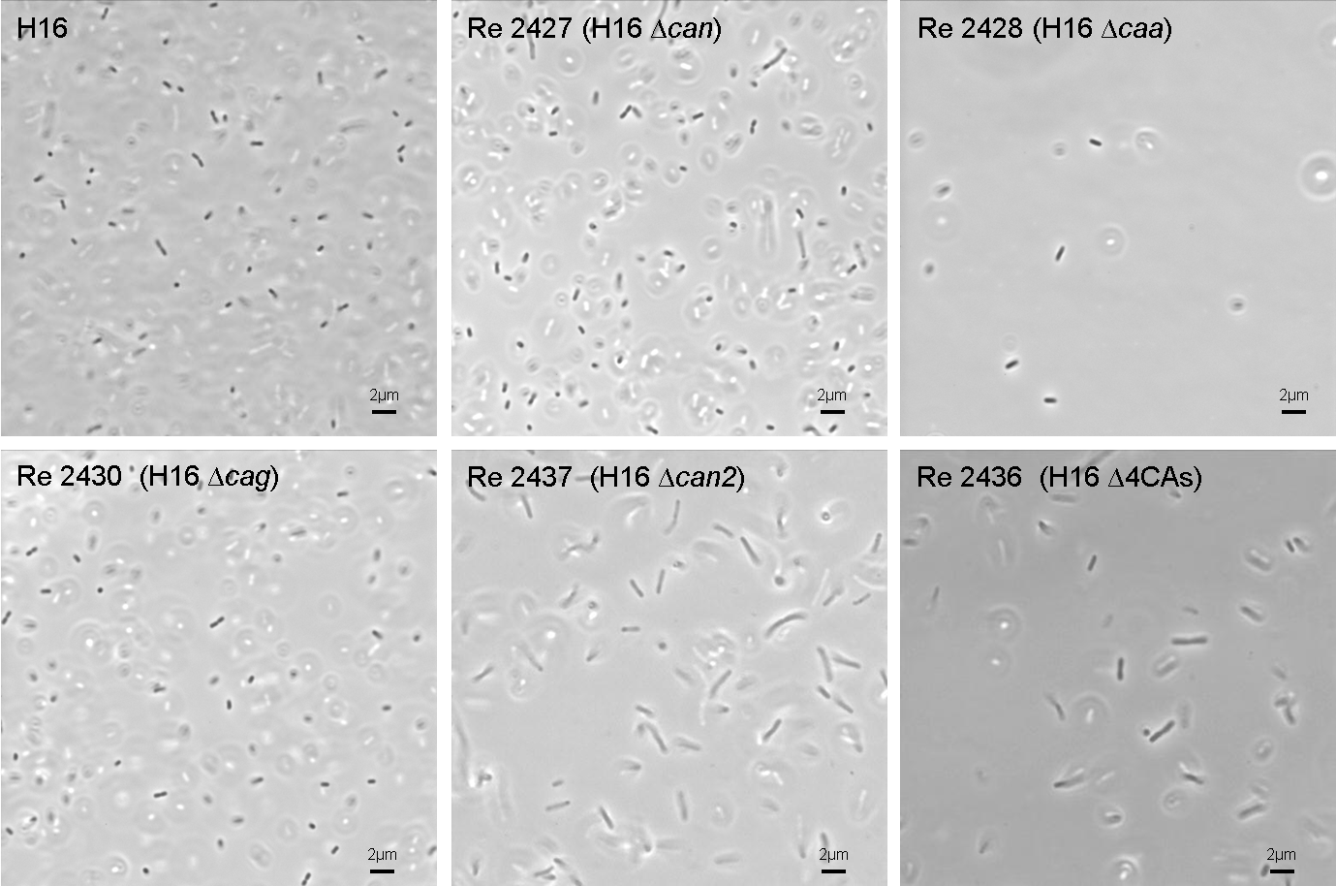


**Additional File 1 Figure S2 Light microscopy of deletion strains.**

Light microscopy images of all CA gene deletion strains and the wild type strain, *R. eutropha* H16 (100X magnification). Scale bar = 2 µm. Strains were grown in TSB media and observed after 24 h of cultivation. Re2427 and Re2436 were cultivated under 10% CO_2_ supplemented environment. Re2437 cells exhibit longer cell morphology, which could be a sign of stress that can be seen in the microscope but unnoticed in the absorbance measurements of the cultures.


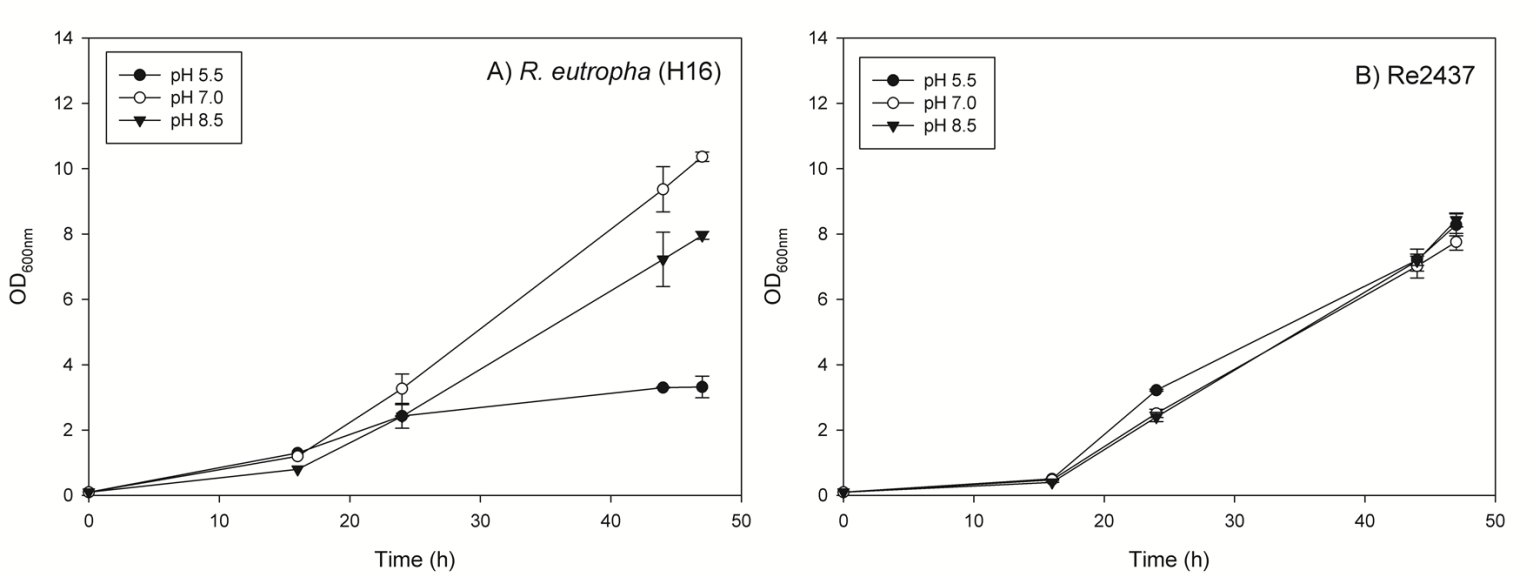


**Additional File 1 Figure S3 Growth of H16 and Re2427 with different initial pH values.**

Growth in minimal media containing 1% fructose (w v^-1^) of **A)** *R. eutropha* (H16) and **B)** Re2437 (H16 Δ*can2*) at different initial medium pH values (5.5, 7.0 and 8.5). The effect of the different pH values can be observed on the wild type but not on the deletion strain, which appears not to sense the effect of the pH change. Values represent average from two replicates with maxima and minima values as error bars.

**
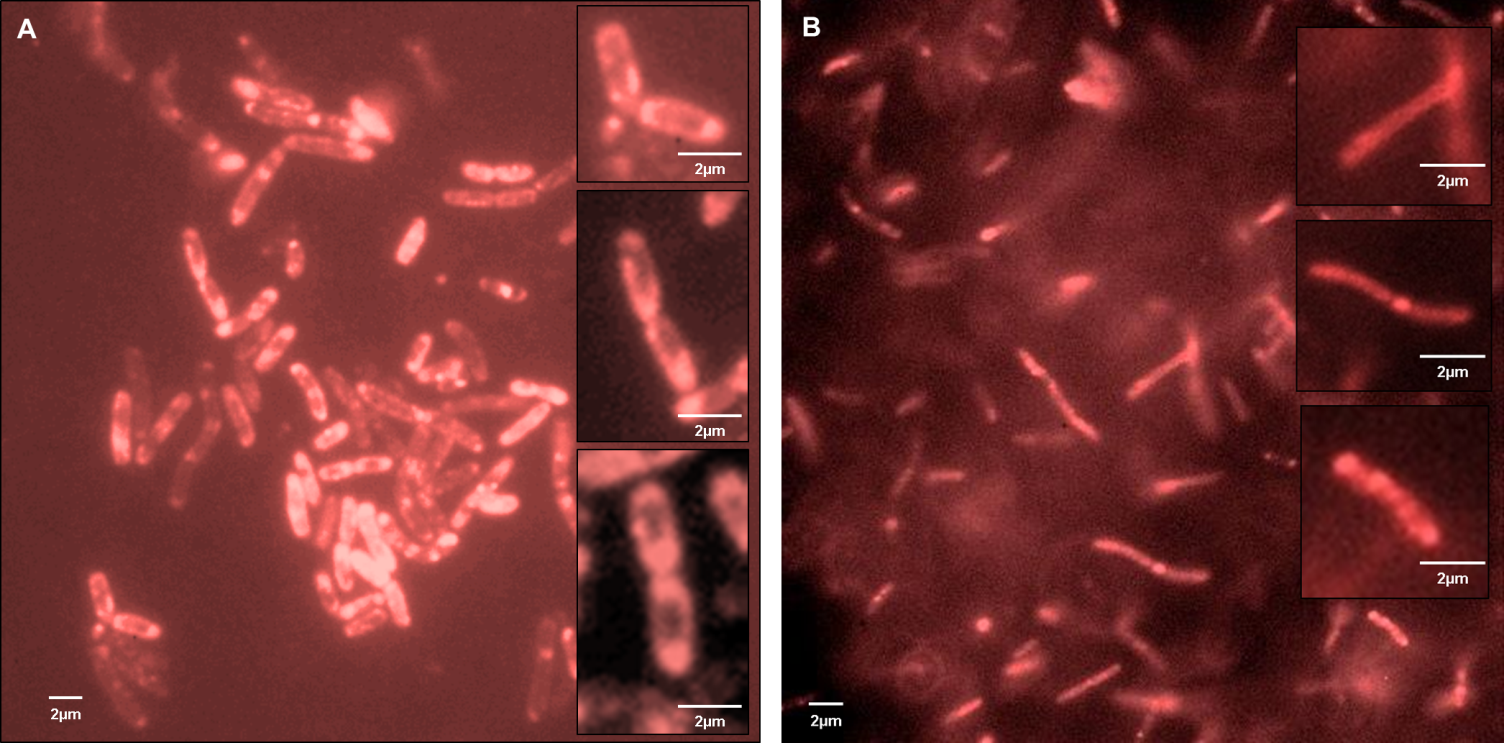
**

**Additional File 1 Figure S4** **Fluorescent microscopy of Caa_RFP fusion protein expressed in Re2061.**

**A)** Fluorescent microscopy (100X magnification) showing *R. eutropha* with constitutively expressed Caa-fused-RFP (Re2061/pCaa_RFP). Details (inset images) show individual cells isolated from the main image where the concentration and localization of the fluorescence near the outer perimeter of the cells were more clearly observed. **B)** Re2061 control expressing RFP alone (Re2061/pRFP). Details show individual cells isolated from the main image where the detected fluorescence is equally spread in the cytosol of the bacterial cells. Scale bar = 2 µm.

**Additional File 1 Table S1** **Oligonucleotide primers used in this study.**

Primers used in the construction of the deletion strains, where the sequences were inserted into pJV7 plasmid. Primers used in the construction of the overexpression plasmids, where sequences were inserted into pBBR1MCS-2. Underlined sequences represent the restriction sites used. The “Gib” indicator denotes that primers were used for Gibson assembly method (Gibson et al. 2009). Some of the oligonucleotide primers listed here were used in more than one construction.

| Gene deleted | Primer | Sequence |
| --- | --- | --- |
| *can*  locus tag H16_A0169 | Can_Del 1 | attGGATCCcactgacgagcaggtcgagcac |
|  | Can_Del 2 | TGGGCGATGGCGTCAGTCATgcgaccctccttgcaggacc |
|  | Can_Del 3 | ATGACTGACGCCATCGCCCAccggacgcacacaccgcttcttc |
|  | Can_Del 4 | attGGATCCcgtcttccaggccgtccagcatc |
| *caa*  locus tag H16_B2403 | Caa_Del 1 | gatggatccgcttgaagtgcagtactggtgc |
|  | Caa_Del 2 | ATCGGCAGCCTGGTGTTCATtgtcgatgcgtcctagtgtt |
|  | Caa_Del 3 | ATGAACACCAGGCTGCCGATaggacggcgcccgatagaagtacc |
|  | Caa_Del 4 | GGATCCctggcagcggtgaagctggtgg |
| *can2*  locus tag H16_B2270 | Can2_Gibs Del 1 | TACGAATTCGAGCTCGGTACCCGGGGATCCgctttcgcgacggcgaatgggtc |
|  | Can2_Del 2 | AGTTGTTCGATGTGATGCATggcggcatccaggcgctac |
|  | Can2_Del 3 | ATGCATCACATCGAACAACTccccatgcatcccttcatccttgg |
|  | Can2_Gibs Del 4 | AAGCTTGCATGCCTGCAGGTCGACTCTAGAcctcgtccttcatggcgaggatg |
| *cag*  locus tag H16_A1192 | Cag_Del 1 | attGAGCTCcagtgccaggttgatggcattgaac |
|  | Cag_Del 2 | CCGAGCTGGTAAAGCGCCATggggtctcctgcacgaaagg |
|  | Cag_Del 3 | ATGGCGCTTTACCAGCTCGGcgaccgcggcggcacttacc |
|  | Cag_Del 4 | attTCTAGAatcgagcccagcgggccatg |
|  | | |
| Gene inserted | Primer | Sequence |
| *can* | Fw can | gataggtacccatatgATGACTGACGCCATCGCCCAGC |
|  | Rv can | cgataagcttggatccTCAGCGGATCGACGC |
| *can2* | Fw can2 | gatagtcgaccatatgATGCATCACATCGAACAACTGC |
|  | Rv can2 | cgataagcttggatccTCAGGGTTCGCAG |
| *caa* | Fw caa | gataggtacccatatgATGAACACCAGGCTGCCG |
|  | Rv caa | cgataagcttggatccCTAGTGGCTGACCTGC |
| *cag* | Fw cag | gataggtacccatatgATGGCGCTTTACCAGCTCGGCG |
|  | Rv cag | cgataagcttctcgagTCAGCCGATCCGCTTGAG |
| *caa*B | Fw caaB | gataggtacccatatgATGGACCCGCACTGGAGCTACA |
|  |  |  |
| *caa*_RFP | Linker Fw | GTGCAGGTCAGCCACTATCCCGCCACCTCCACCTCCatggcgagtagcgaag |
|  | Linker Rv | cttcgctactcgccatGGAGGTGGAGGTGGCGGGATAGTGGCTGACCTGCAC |
| RFP | Rv RFP | ctgcgtcgacttaagcaccggtggagtg |

**Additional File 1 Table S2**

Comparison of CO_2_ hydration activity of different classes of carbonic anhydrases from bacteria and fungi.

| **Class** | **Organism** | **Max SA (CO_2_ hydration)** | **Method used** | **Reference** |
| --- | --- | --- | --- | --- |
| β-CA | *R.eutropha* | ̴20 UCA/mg protein* | Mass spectrometric method ^1^ | Kusian et al. 2002 |
| α CA | *Thiomicrospira crunogena* | 890 + 17 WAU/mg protein | pH monitored electrometrically ^2^ | Dobrinski et al. 2010 |
| β-CA | *T. crunogena* | 3.41 + 0.08 WAU/mg protein | pH monitored electrometrically ^2^ | Dobrinski et al. 2010 |
| β like CA | *T. crunogena* | 1.24 + 0.02 WAU/mg protein | pH monitored electrometrically ^2^ | Dobrinski et al. 2010 |
| α- CA | *Microcoleus chthonoplastes* | 0.238 + 0.01 WAU/mg protein | pH monitored electrometrically ^2^ | Kupriyanova et al. 2007 |
| β-CA | *M. chthonoplastes* | ̴100 WAU/mg protein* | pH monitored electrometrically ^2^ | Kupriyanova et al. 2011 |
| α- CA | *Sulphurihydrogenibium azorense* | ̴ 20000 U/mg protein (80°C)*  ̴2500 U/mg protein (20°C)* | pH monitored using pH-indicator metacresol purple ^3^ | Luca et al. 2013 |
| γ-CA | *Vibrio fischeri* | 1.5 + 0.3 U/mg protein | pH monitored ^4^ | Smith et al. 1999 |
| β-CA | *Staphylococcus aureus* | 1.2 + 0.1 U/mg protein | pH monitored ^4^ | Smith et al. 1999 |
| CA | *Bacillus subtilis* | <0.01 U/mg protein | pH monitored ^4^ | Smith et al. 1999 |
| γ-CA | *Methanosaeta concilii* | 3.0 + 0.4 U/mg protein | pH monitored ^4^ | Smith et al. 1999 |
| β like –CA | *Saccharomyces cerevisiae* | ̴6900 U/mg protein | Mass spectrometric method ^1^ | Amoroso et al. 2005 |
| α- CA | *Cryptococcus neoformans* | ̴20 WAU* | pH monitored electrometrically ^2^ | Mogensen et al. 2006 |
| α- CA | *R. eutropha* | 422.32 ± 97.05 EU/mg protein (0°C) | pH monitored using pH standard phenol red ^5^ | This study |
| β-CA | *R. eutropha* | 59.5 ± 15.35 EU/mg protein (0°C) | pH monitored using pH standard phenol red ^5^ | This study |
| β-CA | *R. eutropha* | 157.94 ± 41.16 EU/mg protein (0°C) | pH monitored using pH standard phenol red ^5^ | This study |
| γ-CA | *R. eutropha* | 138.19 ± 61.27 EU/mg protein (0°C) | pH monitored using pH standard phenol red ^5^ | This study |

*- Data extracted from a graph.

1. Sültemeyer et al. (1998) Mass spectrometric method based on loss of ^18^O from doubly labeled ^13^C^18^O to water.

2. Wilburg and Anderson (1948) pH monitored from 8.0 to 7.0 electrometrically. Reaction kept at 4°C.

3. Chirica et al. (1997) pH monitored using pH-indicator Taps/NaOH/metacresol purple monitored at 578 nm. Reaction kept at 25°C.

4. Smith et al. (1999) pH monitored from 7.8 to 7.0. Reaction kept at 23°C or 55°C.

5. Sundaram et al. (1986) pH monitored using pH standard phenol red. Reaction kept at 0°C.

**References from Additional File 1 which do not appear in main text references**

Amoroso G, Morell-Avrahov L, Müller D, Klug K, Sültemeyer D (2005) The gene NCE103 (YNL036w) from *Saccharomyces cerevisiae* encodes a functional carbonic anhydrase and its transcription is regulated by the concentration of inorganic carbon in the medium. Molec Microbiol 56:549–558

Chirica LC, Elleby B, Jonsson BH, Lindskog S (1997) The complete sequence, expression in *Escherichia coli*, purification and some properties of carbonic anhydrase from *Neisseria gonorrhoeae*. Eur J Biochem 244:755–760

Gibson DG, Young L, Chuang RY, Venter JC, Hutchison CA 3rd, Smith HO (2009) Enzymatic assembly of DNA molecules up to several hundred kilobases. Nat Methods 6:343–345

Kupriyanova EV, Sinetova MA, Markelova AG, Allakhverdiev SI, Los DA, Pronina NA (2011) Extracellular β-class carbonic anhydrase of the alkaliphilic cyanobacterium *Microcoleus chthonoplastes*. J Photochem Photobiol B 103:78–86

Luca VD, Vullo D, Scozzafava A, Carginale V, Rossi M, Supuran CT, Capasso C (2013) An α-carbonic anhydrase from the thermophilic bacterium *Sulphurihydrogenibium azorense* is the fastest enzyme known for the CO2 hydration reaction. Bioorg Med Chem 21:1465–1469

Mogensen EG, Janbon G, Chaloupka J, Steegborn C, Fu MS, Moyrand F, Klengel T, Pearson DS, Geeves MA, Buck J, Levin LR, Mühlschlegel FA (2006) *Cryptococcus neoformans* senses CO2 through the carbonic anhydrase Can2 and the adenylyl cyclase Cac1. Eukariotic Cell 5:103–111

Sültemeyer D (1998) Carbonic anhydrasein eukaryotic algae: characterization, regulation, and possible function during photosynthesis. Can J Bot 76:962–972

Wilbur KM, Anderson NG (1948) Electrometric and colorimetric determination of carbonic anhydrase. J Biol Chem 176:147–154
